# A New Model to Predict Optimum Conditions for Growth of 2D Materials on a Substrate

**DOI:** 10.3390/nano9070978

**Published:** 2019-07-05

**Authors:** Yu-Peng Liu, Bo-Yuan Ning, Le-Cheng Gong, Tsu-Chien Weng, Xi-Jing Ning

**Affiliations:** 1 Institute of Modern Physics, Fudan University, Shanghai 200433, China; 2 Applied Ion Beam Physics Laboratory, Fudan University, Shanghai 200433, China; 3 Center for High Pressure Science & Technology Advanced Research, Shanghai 202103, China

**Keywords:** 2D materials, free energy, partition function, graphene, γ-graphyne, silicene

## Abstract

Deposition of atoms or molecules on a solid surface is a flexible way to prepare various novel two-dimensional materials if the growth conditions, such as suitable surface and optimum temperature, could be predicted theoretically. However, prediction challenges modern theory of material design because the free energy criteria can hardly be applied to this issue due to the long-standing problem in statistical physics of the calculations of the free energy. Herein, we present an approach to the problem by the demonstrations of graphene and γ-graphyne on the surface of copper crystal, as well as silicene on a silver substrate. Compared with previous state-of-the-art algorithms for calculations of the free energy, our approach is capable of achieving computational precisions at least 10-times higher, which was confirmed by molecular dynamics simulations, and working at least four orders of magnitude faster, which enables us to obtain free energy based on ab initio calculations of the interaction potential instead of the empirical one. The approach was applied to predict the optimum conditions for silicene growth on different surfaces of solid silver based on density functional theory, and the results are in good agreement with previous experimental observations.

## 1. Introduction

Since graphene was obtained in 2004 [1], 2-dimensional (2D) materials have developed a wide interest all over the world. Due to the ultrathin thickness, the materials exhibit unique electrical, mechanical, and thermal properties, which inspires explorations of other more 2D materials. So far, dozens of 2D materials have been prepared experimentally, including the graphene family (e.g., graphdiyne [2], silicene [3], germanene [4], borophene [5], phosphorene [6], bismuthene [7]), Ⅲ-nitrides [8,9], Ⅲ-bismides [10], transition metal dichalcogenides [11], metal carbides [12], and the like, yet it remains a problem to prepare high-quality 2D material with larger sizes. Although a few kinds of 2D materials such as graphene and MoS_2_ [13] can be obtained by exfoliating corresponding layered bulk materials, others, such as silicene [3,14,15,16,17,18,19,20,21,22], cannot be produced in this way because there exists no corresponding layered bulk material in nature. Vapor deposition of atoms on a substrate should be much more flexible and has been applied for preparing various 2D materials of large scale [3,23]. However, much time and effort have to be paid to explore the growth conditions because the surface structure and the temperature of the substrate both have significant effects on the growth of 2D materials. As an example for preparing silicene by deposition of Si atoms on a silver substrate, the growth on the (001) surface only produces a “complex” superstructure without clear symmetry [24], and on the (110) surface, silicon nanoribbons (NRs) form along the $\left[\bar{1}10\right]$ direction with a honeycomb structure [25,26], while on the (111) surface, a continuous graphene-like 2D honeycomb arrangement of silicon atoms, silicene, can be obtained [3,27]. It is notable that the silicene can grow only when temperature of the substrate is kept between 220 and 250 °C, showing quite a significant effect of the temperature on the growth. 

Clearly, many experimental efforts can be saved for preparing the desired 2D materials by vapor deposition of the atoms if the suitable surface of a substrate and the optimum temperature can be precisely predicted. The predictions might be made in principle by calculations of the free energy, but the calculation for condensed matter has been an open problem since statistical physics was born at the end of 19th Century. In the past 30 years, substantial progress has been made in the calculations of the partition function (PF) for condensed matter, from which the free energies, as well as other thermodynamic state functions can be obtained. Among the advanced methods including parallel tempering [28], umbrella sampling [29], metadynamics [30], Wang–Landau sampling [31], and nested sampling [32], an algorithm of nested sampling (ANS) developed by Do et al [33] may be the state-of-the-art and is able to compute PF for a condensed system composed of hundreds of atoms if empirical pairwise potentials are applied for the interaction of atoms. In ANS, all the atoms in the system are moved artificially in real space so as to generate enough configurations, usually on the order of 10^8^ for a system of hundreds of atoms, with the total potential energy of each configuration being calculated, which will cost too many computer hours if the interactions between atoms are described by quantum mechanics or by an empirical many-body potential, such as the Brenner function [34] for carbon atoms of graphene, instead of a pairwise force field. Moreover, when the algorithm is applied to calculate the PF of a given 2D material, such as a graphene sheet with a perfect hexagons structure, artificially moving the C atoms in real space may produce structures approaching those of graphyne or others, so the final obtained PF may not be related uniquely to the graphene sheet. Obviously, artificial constraints must be applied in ANS to move the atoms so that the generated molecular configurations are closely related to the structure of the given 2D materials, which will result in large uncertainties of the PF. In such a case, the difference of the free energy derived from the PF between two different 2D structures will depend too much on the artificial constraints, leading to the failure of the free energy criteria to judge which 2D structure would be more favorable.

In the present work, a direct integral approach (DIA) [35] was developed to calculate the PF (or free energy) of 2D materials and was demonstrated by graphene and γ-graphyne on a Cu substrate, as well as silicene on a silver substrate. In order to test the accuracy of the approach, the empirical many-body interaction function, Brenner potential [34], and Tersoff potential [36] were employed to calculate the PF of graphene (or γ-graphyne) and silicene, respectively, and the same potential was used in MD simulations to produce internal energy to be compared with the one derived from the PF. The high efficiency of DIA enables us to obtain the PF of the systems of 510 carbon atoms (or 336 Si atoms) in about one hour on a desktop computer, and the relative error between the derived internal energies and the ones of the MD simulations for the system at temperatures from 100–1300 K are smaller than 0.03%, which is far beyond the precision (~10%) usually achieved by ANS [37]. Certainly, the calculation precision of free energy cannot be determined directly because MD simulations can hardly produce free energy [38]. The calculations with DIA show that the free energy of graphene is always smaller than that of γ-graphyne in the temperature range from 100–3000 K, which is in agreement with the experimental fact that the growth of graphene under such conditions is much easier than that of γ-graphyne. Furthermore, DIA was applied to search for the optimum conditions for silicene growth on a Ag substrate based on density functional theory (DFT), and the results were in good agreement with previous experimental observations.

## 2. Theoretical Method

**Figure 1:**
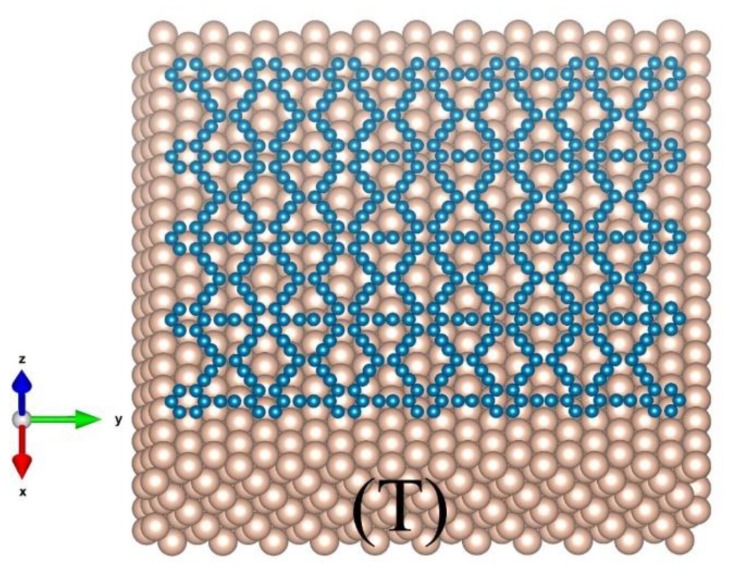
Schematic of a piece of 2D material of *N* atoms (blue color) lying on the surface of a substrate of *M* atoms (golden color) at temperature *T*.

The model for a 2D materials on a substrate is shown in Figure 1, where the substrate of *M* atoms is treated as a thermal bath at temperature *T*. For calculations of the PF for the 2D materials of *N* atoms, the total potential is expressed as:

(1) $$U\left(x^{3N},X^{3M}\right)=U_{2D}\left(x^{3N}\right)+V\left(x^{3N},X^{3M}\right),$$

where $U_{2D}$ is the potential energy of the 2D material with the coordinates of the atoms denoted by $x^{3N}\left(x_{1},x_{2}\ldots x_{3N}\right)$ and *V* is the interaction potential between the 2D material and the substrate with its atoms denoted by $X^{3M}\left(X_{1},X_{2}\ldots X_{3M}\right)$. 

The PF of the canonical ensemble for 2D materials can be expressed as:

(2) $$Z=\frac{1}{N!}{\left(\frac{2\pi m}{\beta h^{2}}\right)}^{\frac{3N}{2}}Q,$$

where *β* = 1/$k_{B}T$ with *k_B_* the Boltzmann factor and *Q* is the configurational integral:

(3) $$Q=\int dx^{3N}\exp\left[-\beta U\left(x^{3N},X^{3M}\right)\right].$$

In order to solve the 3*N*-fold *Q* integral, the sense of the integral is reinterpreted as follows [35]. Traditionally, a 1D integral $I_{1D}=\int_{a}^{b}f\left(x\right)dx$ is interpreted as the sum of an infinite number of rectangles with area $A_{i}=f\left(x_{i}\right)\Delta x$, i.e., $I_{1D}=\lim_{\Delta x\to 0}\sum_{i}A_{i}$. From another angle, the length of the 1D element Δx at $x_{i}$ is modulated by $f\left(x_{i}\right)$ to be a new length element $\Delta x_{i}^{\prime }$ = $f\left(x_{i}\right)\Delta x$ and $I_{1D}=\sum_{i}\Delta x_{i}^{\prime }.$ In other words, the 1D integral is a summation of length elements instead of area elements and equals an effective length of |b−a|. Similarly, a 2D integral $I_{2D}=\int_{0}^{a}\int_{0}^{b}dxdyf\left(x,y\right)$ equals an effective area of a·b because the area element *ds* = *dxdy* is enlarged (or shrunk) by *f*(*x*,*y*), giving rise to an effective area element $ds^{\prime }=f\left(x,y\right)dxdy.$ Followed by this notion, an *N*-fold integral $I_{ND}=\int_{0}^{a_{1}}\int_{0}^{a_{2}}\ldots \int_{0}^{a_{N}}dx_{1}dx_{2}\ldots dr_{N}f(x_{1},x_{2}\ldots x_{N})$ equals an effective volume of $a_{1}\cdot a_{2}\ldots a_{N}.$

When the integrand $f\left(x_{1},x_{2}\ldots x_{N}\right)$ is in a form of $\exp[-U(x_{1},x_{2}\ldots x_{N}$)] with *U* ($x_{1},x_{2}\ldots x_{N}$) being positive definite within the entire integral domain and having the minimum at the origin (U(0)=0), the effective length of $a_{i}$ is defined as [35]:

(4) $$a_{i}^{\prime }=\int_{0}^{a_{i}}\exp[-U(0\ldots x_{i}\ldots 0)]dx_{i},\left(i=1,2\ldots N\right),$$

and the effective volume approximates to a product $\prod_{i=1}^{N}a_{i}^{\prime }$, i.e.,

(5) $$I_{ND}≅\prod_{i=1}^{N}a_{i}^{\prime }.$$

For the proof of Equation (5), we consider a 2D integral:

(6) $$I_{2D}=\int_{0}^{a}dx\int_{0}^{b}dye^{-U\left(x,y\right)},$$

where *U*(*x*, *y*) is positive definite within the integral domain, 0 ≤ *x* ≤ *a*, 0 ≤ *y* ≤ *b*, on which we define a map of *x* and *y*:

$$X^{\prime }\left(x\right)=\int_{0}^{x}e^{-U\left(\xi ,0\right)}d\xi ,$$

(7) $$Y^{\prime }\left(x\right)=\int_{0}^{y}e^{-U\left(\xi ,0\right)}d\xi ,$$

Therefore, $X^{\prime }\left(0\right)=0$, $Y^{\prime }\left(0\right)=0$. The effective length of *a* and *b* is defined by:

$$a^{\prime }=\int_{0}^{a}e^{-U\left(\xi ,0\right)}d\xi ,$$

(8) $$b^{\prime }=\int_{0}^{b}e^{-U\left(0,\xi \right)}d\xi .$$

Inserting Equation (7) into Equation (6) yields:

(9) $$I_{2D}=\int_{0}^{a^{\prime }}dX^{\prime }\int_{0}^{b^{\prime }}dY^{\prime }e^{-F\left(X^{\prime },Y^{\prime }\right)},$$

with:

(10) $$F\left(X^{\prime },Y^{\prime }\right)=U\left(x\left(X^{\prime }\right),y\left(Y^{\prime }\right)\right)-U\left(x\left(X^{\prime }\right),0\right)-U\left(0,y\left(Y^{\prime }\right)\right),$$

which can be expanded in Taylor series as:

(11) $$F\left(X^{\prime },Y^{\prime }\right)=F\left(0,0\right)-\frac{\partial F}{\partial X^{\prime }}{|}_{0}\Delta X^{\prime }-\frac{\partial F}{\partial Y^{\prime }}{|}_{0}\Delta Y^{\prime }.$$

If U(x,y) has a minimum at the origin (*x* = 0, *y* = 0), i.e., U(0,0) = 0, then F(0,0)=0, $\frac{\partial F}{\partial X^{\prime }}{|}_{0}=\frac{\partial F}{\partial Y^{\prime }}{|}_{0}$ = 0, and thus, the value of $F\left(X^{\prime },Y^{\prime }\right)$ is close to zero in the neighborhood of (0, 0). Since U(x, y) is positive definite, $a^{\prime }$ and $b^{\prime }$ are small, and the integral domain of Equation (9) is a small area around the origin, we obtain that:

(12) $$I_{2D}≃a^{\prime }b^{\prime }.$$

The proof can be easily extended to an N-dimensional integral, $I_{ND}=\prod_{i=1}^{N}\int_{0}^{a_{i}}dq_{i}\exp[-U\left(q_{1},q_{2}\ldots q_{N}\right)$], as long as the function $U\left(q_{1},q_{2}\ldots q_{N}\right)$ is positive definite and has a minimum at the origin (U(0) = 0).

For the 3*N*-fold integral of Equation (3), although the integrand is of the same form as required by Equation (5), it may not be positive definite or have no minimum at the origin. Letting $q^{3N}=\{q_{1},q_{2}\ldots q_{3N}\}$ be the coordinates of particles in the state of the lowest potential energy $U_{0}$, we introduce a function:

(13) $$U^{\prime }\left(x^{\prime }{}^{3N},X^{3M}\right)=U\left(x^{3N},X^{3M}\right)-U_{0},$$

with $x_{i}^{\prime }=x_{i}-q_{i}$. By inserting Equation (13) into Equation (3), we obtain:

(14) $$Q=e^{-\beta U_{0}}\int x^{3N}\exp\left[-\beta U^{\prime }\left(x^{\prime }{}^{3N},X^{3M}\right)\right].$$

Clearly, $U^{\prime }\left(x^{\prime }{}^{3N},X^{3M}\right)$ is positive definite within all the integral domains and has the minimum at the origin ($U^{\prime }\left(0,X^{3M}\right)=0)$. According to Equation (5), the integral in Equation (14) equals an effective 3*N*-dimensional volume,

(15) $$Q=e^{-\beta U_{0}}\prod_{i=1}^{3N}L_{i},$$

where the effective length $L_{i}$ on the *i*^th^ degree of freedom is defined as:

(16) $$L_{i}=\int e^{-\beta U^{\prime }\left(0\ldots x_{i}^{\prime }\ldots 0,X^{3M}\right)}dx_{i}^{\prime }.$$

For a 2D material sheet on a substrate (Figure 1), however, the effective length $L_{z}$ might be different from $L_{y}$ or $L_{x}$, and the edge atoms ($N_{1}$) should have different effective lengths from the ones of the atoms ($N_{2}$) in the center region. In such a case, Equation (15) turns into:

(17) $$Q=e^{-\beta U_{0}}[L_{x}^{1}L_{y}^{1}L_{z}^{1}{]}^{N1}\cdot [L_{x}^{2}L_{y}^{2}L_{z}^{2}{]}^{N2}.$$

To obtain the effective lengths, the first step is to find the most stable structure of the 2D materials with the lowest potential $U_{0}$, which can be accomplished by various well-developed global optimization algorithms [39,40,41,42] or the dynamic damping method [43,44]. Starting from the most stable structure, one atom in the center region (or in the edge region) is moved step by step in one of the degrees of freedom, such as the X-axis, while the Y- and Z-coordinates and all other atoms are kept fixed to determine $U^{\prime }\left(0\ldots x_{i}^{\prime }\ldots 0,X^{3M}\right)$ for calculating $L_{x}^{1},\;L_{y}^{1},\;L_{z}^{1}$ (or $L_{x}^{2},\;L_{y}^{2},\;L_{z}^{2}$). Clearly, it is an easy task for traditional ab initio algorithms and recently-developed DFT [45,46]. 

The computational cost of DIA and ANS [33] is determined by the number of times of the calculations of the potential energy. For a system consisting of hundreds of atoms [33,37,47,48], ANS partitions configuration space into at least $10^{3}$ subdivisions, and in each subdivision, more than $3\times 10^{4}$ configurations should be randomly produced to be calculated for the total potential energy, and the same program of ANS must be repeatedly run dozens of times to produce average results because of the fluctuations of the Monte Carlo algorithm in ANS. Therefore, the times for ANS calculating the potential are more than $3\times 10^{8},$ while the times needed by DIA can be fewer than $1\times 10^{4}$ because $U^{\prime }\left(0\ldots x_{i}^{\prime }\ldots 0\right)$ in Equation (9) can be well determined with a step of 0.001 Å for changes of $x_{i}$ in a range of about 1 Å, indicating that DIA works at least four orders of magnitude faster than ANS.

## 3. Demonstrations of the Model

### 3.1. Graphene and γ-Graphyne on a Cu Substrate

**Figure 2:**
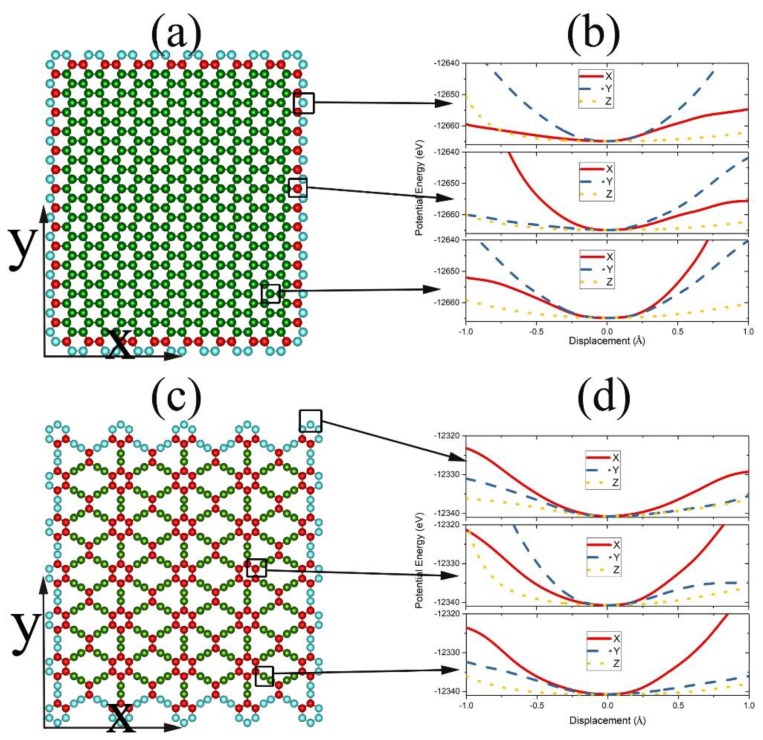
Top view of a graphene sheet (**a**) and a γ-graphyne sheet (**c**) on a Cu (111) substrate. The potential energy $U^{\prime }$ (**b**,**d**) felt by a C atom moving along the X-, Y-, or Z-axis depends on the specific surrounding of the C atom.

The approach developed in the last section was first demonstrated by calculating the PF for a piece of graphene (Figure 2a) or γ-graphyne (Figure 2c) sheet of 510 C atoms on the (111) surface of a Cu substrate of 2640 atoms located in perfect fcc lattices. Brenner potential was employed to characterize C atoms’ interaction with the empirical parameters taken from [34], and $V\left(x^{3N},X^{3M}\right)$ was taken as the summation of the Lennard–Jones (L-J) potential $f\left(r\right)=4\varepsilon \left(\frac{\sigma^{12}}{r^{12}}-\frac{\sigma^{6}}{r^{6}}\right)$ for the pairwise interaction between a C and a Cu atom with ε = 0.0168 eV, σ=2.2 Å [49].

The system was cooled below 0.01 K by a damping method [43,44] to determine the lowest energy $U_{0}$ and the most stable structure. According to the configuration (Figure 2a,c), the C atoms were divided into a center group and an edge group, and for each of the atoms with different surroundings, one of its coordinates (such as $x^{\prime }$) was changed step by step with an interval of 0.001 Å, while its $y^{\prime }$ and $z^{\prime }$ coordinates and all other atoms were kept fixed to record $U^{\prime }\left(0\ldots x_{i}^{\prime }\ldots 0,X^{3M}\right)$, as shown in Figure 2b and d for graphene and γ-graphyne, respectively. $U^{\prime }$ for the center atom is indeed different from that of the edge atoms, and for the same atom, $U^{\prime }$ along one coordinate axis is also different from that along the other two, so the configuration integral was calculated as:

(18) $$Q=e^{-\beta U_{0}}[L_{X}^{1}L_{Y}^{1}L_{Z}^{1}{]}^{N1}\cdot [L_{X}^{2}L_{Y}^{2}L_{Z}^{2}{]}^{N2}\cdot {\left[L_{X}^{3}L_{Y}^{3}L_{Z}^{3}\right]}^{N3}.$$

By applying Equation (2) and $E=-\frac{\partial }{\partial \beta }\ln Z$, the internal energy ($E_{PF}$) was obtained through:

(19) $$E_{PF}=\frac{3}{2}Nk_{B}T+\frac{k_{B}T^{2}}{Q}\frac{\Delta Q}{\Delta T},$$

with temperature difference ΔT = 0.1 K.

In order to test the accuracy, a common procedure for MD simulations of a canonical ensemble [43,44] was employed to produce the internal energy of the 2D materials in contact with a thermal bath at a given temperature *T*. Specifically, the atoms of the substrate (Figure 1) were fixed while the C atoms moved according to classical equations of motion, which were solved by the Verlet algorithm with a time step of 0.2 fs. Within the first 400 fs, all the carbon atoms were assigned velocities every 40 fs according to the Maxwell velocity distribution at temperature *T*, and then, the internal energy ($E_{MD}$) and the temperature were recorded every 30 fs to perform the average over 100 records. 

**Figure 3:**
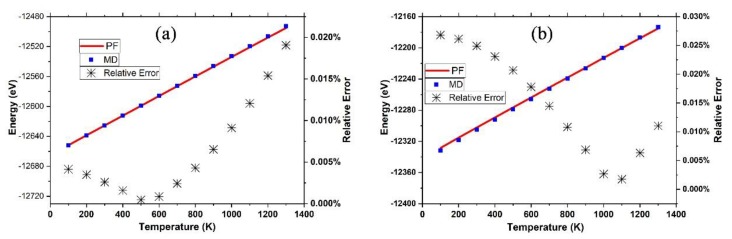
The internal energy as the function of temperature derived from the partition function (PF) (red line) or MD simulation (blue square) for the graphene sheet (**a**) and the γ-graphyne sheet (**b**), where the relative errors $\frac{\left|E_{PF}-E_{MD}\right|}{\left|E_{MD}\right|}\times 100\%$ (black stars) are characterized at the right vertical axis.

As shown in Figure 3, the internal energy ($E_{PF}$) derived from the PF was in excellent agreement with that ($E_{MD}$) obtained from the MD simulations with a relative standard deviation of 0.00002%, which is too small to be shown in Figure 3. In the temperature range from 100 K to 1300 K, the relative error ($\frac{\left|E_{PF}-E_{MD}\right|}{\left|E_{MD}\right|}\times 100\%$) as below 0.03%, and only 0.0005% and 0.002% for graphene at 500 K and γ-graphyne at 1100 K, respectively. We cannot make the comparisons of the precisions between DIA and that of ANS [33] because the implementation of ANS costs too many computer hours for this system and the followed ones in this work. Nevertheless, the internal energy errors for ANS applied in crystal argon systems of 500 atoms characterized by the L-J potential function, which is much simpler than the many-body Brenner-function used here, were above 10% [37], which was much larger than the above ones.

It is notable that the dependence of internal energy (*E*) on temperature is nearly linear, indicating that $E=U_{0}+BNk_{B}T$, where *B* is a constant. According to the statistical physics, the constant *B* equals three for 3D crystal atoms with harmonic coordinates. However, for the graphene and γ-graphyne sheet, the constant *B* equals 2.97 and 2.90, respectively, showing that the C atoms are not in the motion of harmonics.

**Figure 4:**
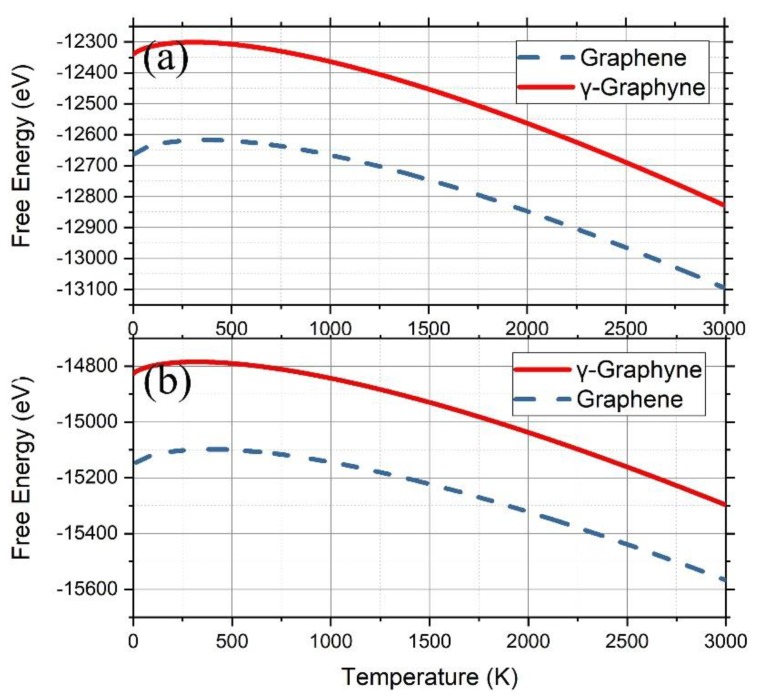
Free energy derived from the PF of graphene (blue dashed lines) and γ-graphyne (red solid lines) on the substrates of Cu [111] (**a**) and Ni [111] (**b**).

Applying $F=-k_{B}T\ln Z,$ the free energy of graphene and γ-graphyne on Cu (111) was calculated (Figure 4a), showing that the free energy of graphene is always smaller than that of γ-graphyne in the temperature range from 100 K to 3000 K. The difference at 100 K, 321 eV, decreased gradually down to 267 eV with the temperature increasing up to 3000 K, indicating that graphene should be more easily grown than γ-graphyne on a Cu (111) surface via depositing C atoms. We also calculated the free energy on a Ni (111) surface (Figure 4b) using the same method and found that graphene still owned smaller free energy than γ-graphyne, although the difference at 100 K, 318 eV, became gradually small with the temperature. These results are consistent with previous experimental observations [50,51].

### 3.2. Silicene on Silver Substrate 

**Figure 5:**
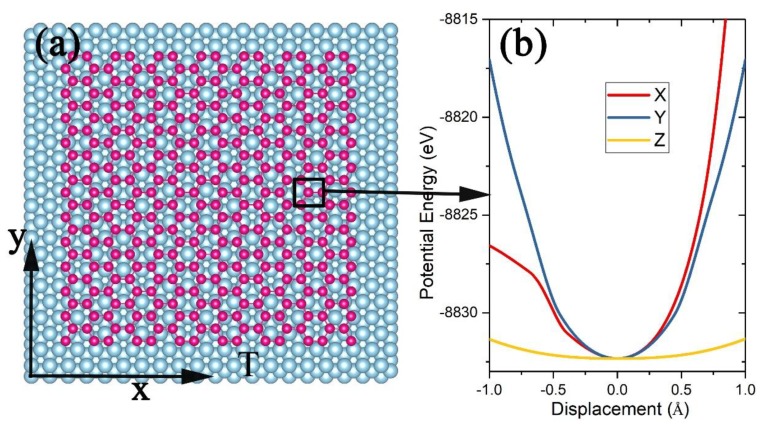
(**a**) Schematic of a piece of silicene of N atoms (red balls) lying on the surface of a Ag(111) substrate of M atoms (blue balls) at temperature T and (**b**) the potential energy felt by a Si atom (shown in the black box) moving along the X-, Y-, or Z-axis.

For a piece of silicene sheet of 336 Si atoms on the (111) surface of an Ag substrate with 2640 Ag atoms located in perfect fcc lattices (Figure 5a), the Tersoff potential was employed to describe the interactions between Si atoms with the parameters taken from [36], and the interaction between the Si atoms and the Ag atoms as described by a Morse pairwise function [52]. The system was cooled down to 0.01 K by a damping method [43,44] to determine the lowest energy $U_{0}$ and the most stable structure. Although $U^{\prime }\left(0\ldots x_{i}^{\prime }\ldots 0,X^{3M}\right)$ felt by an atom *i* located at the edges of the silicene sheet should be different from the one felt by an atom in the center region, the edge atoms are much fewer than the center atoms, so we only calculated $U^{\prime }$ felt by a center atom (Figure 5b) to determine the effective length $L_{x}$, $L_{y}$, and $L_{z}$, and the configuration integral approximates to:

(20) $$Q=e^{-\beta U_{0}}{\left[L_{x}L_{y}L_{z}\right]}^{N},$$

according to Equation (2) and $E=-\frac{\partial }{\partial \beta }\ln Z$, the internal energy ($E_{PF}$) can be obtained through Equation (19) with ΔT = 0.1 K.

A classical MD simulation was performed by using the Large-scale Atomic/Molecular Massively Parallel Simulator [53] program to produce the internal energy of the system with the same potentials [36,52] as those for calculations of the PF. Specifically, the time step as set as 0.1 fs, and the velocities of the silicon atoms were assigned according to a time integration on Nose–Hoover equations of motion to keep the system at a given temperature. Then, the internal energies ($E_{MD}$) and the temperature were recorded every 30 fs to perform the average over 100 records.

**Figure 6:**
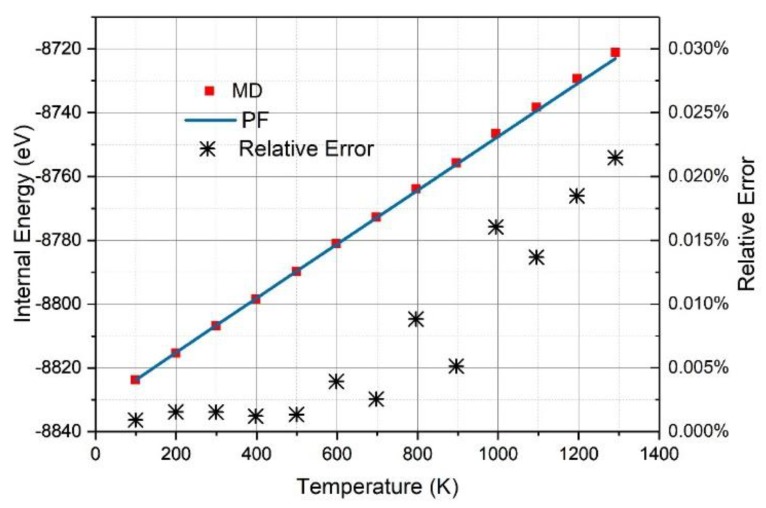
The internal energy as a function of temperature derived from the PF (blue line) or MD simulation (red square), where the relative errors $\frac{\left|E_{PF}-E_{MD}\right|}{\left|E_{MD}\right|}\times 100\%$ (black stars) are characterized at the right vertical axis.

As shown in Figure 6, the internal energy ($E_{PF}$) derived from the PF was in excellent agreement with that ($E_{MD}$) obtained by the MD simulations with a relative standard deviation of 0.008%, which is too small to be shown in Figure 6. For temperatures lower than 500 K, the relative error ($\frac{\left|E_{PF}-E_{MD}\right|}{\left|E_{MD}\right|}\times 100\%$) was only 0.001% and gradually increased up to 0.021% for 1300 K. It may be expected that the relative error would get smaller if the effective length $L_{i}$ of the edge atoms was calculated, instead of replacing $L_{i}$ with the one of the center atom, to calculate Q by Equation (11). 

## 4. Conditions for Silicene Growth on a Ag Substrate

In order to search for the optimum conditions for given silicene grain growth on a Ag substrate, we calculated the FE ($F=-k_{B}T\ln Z$) by DIA with the potential $U^{\prime }$ determined by DFT, which were performed by the Vienna ab initio Simulation Package based on local density approximation. The kinetic-energy cutoff for the plane-wave basis set was 400 eV, and the Brillouin zone was sampled with (2×2×1) *k*-points. 

**Figure 7:**
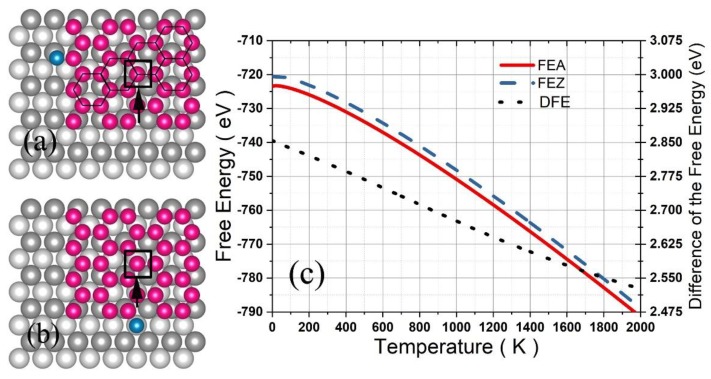
A silicene grain of four silicon hexagons on the Ag (110) surface with a deposited Si atom adhered to the zigzag (**a**) or armchair edge (**b**). The free energy for the zigzag adherence and armchair adherence and the difference are shown in (**c**).

Considering that silicene can grow on a Ag (110) surface by deposition of Si atoms, we calculated the FE of a silicene grain consisting of 35 Si atoms in hexagon arrangement with one Si atom adhered to the zigzag (Figure 7a) or armchair edge (Figure 7b) on the surface of four atomic layers with each containing 40 Ag arranged in an 8×5 fcc supercell. According to thermodynamics, if the free energy for the zigzag adherence (FEZ) equals the one for the armchair adherence (FEA), then both the zigzag and armchair edge of the grain are able to grow larger. Otherwise, the future Si atoms deposited on the Ag surface would more favorably arrive at the zigzag (or armchair) edge if FEZ is lower (or larger) than FEA, resulting in development of only one of the edges to form a band (or nanoribbon).

For calculations of FEZ and FEA, the system was optimized firstly with the bottom layer of the Ag substrate fixed, and then, the adhered Si atom (and one of the Si atoms denoted by a square in Figure 7a,b in the center of the silicene) was moved along the X, Y, and Z direction step by step with an interval of 0.1 Å to produce $U^{\prime }$ for calculating the effective length $l_{x}$, $l_{y}$, and $l_{z}$ (or the $L_{x}$, $L_{y}$, and $L_{z}$) via Equation (9), and finally, the configurational integral was obtained by:

(21) $$Q=e^{-\beta U_{0}}\left(l_{x}l_{y}l_{z}\right){\left(L_{x}L_{y}L_{z}\right)}^{N_{0}},$$

where $N_{0}$ = 35, the number of Si atoms except for the adhered Si atom.

As shown in Figure 7c, FEZ and FEA decreased significantly with the temperature increasing up to 2000 K, while the difference DFE (= FEZ-FEA) decreased gradually from 2.854 eV for 0 K down to 2.526 eV for 2000 K. According to the thermodynamics, if a Si atom deposited on this surface has enough time to wander between the two edges, then it will finally locate at the armchair edge because of the lower FE, and five similar Si atoms will form an armchair edge instead of a zigzag one. As a result, the armchair edge progresses row by row, and the initial silicene grain eventually grows into a nanoribbon along the [$\bar{1}10$] direction with a width of about 1.6 nm, which is the exact observation in the previous experiment [25,26].

**Figure 8:**
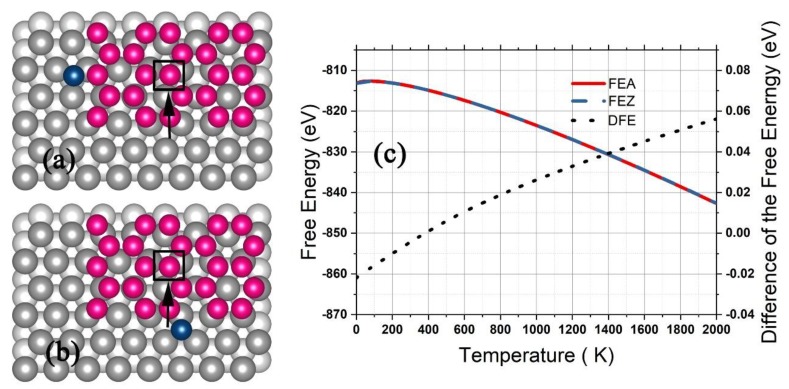
A silicene grain of four silicon hexagons on the Ag (111) surface with a deposited Si atom adhered to the zigzag (**a**) or armchair edge (**b**). The free energy for the zigzag adherence and armchair adherence and the difference are shown in (**c**).

For the growth on the Ag (111) surface, a silicene grain of 25 Si atoms arranged in hexagons with a Si atom adhered at the zigzag edge (Figure 8a) or the armchair edge (Figure 8b) was placed on an Ag substrate of four (111) layers with each consisting of 48 Ag atoms in a (6 × 8) supercell, and the geometry was optimized with the bottom layer of the Ag substrate fixed. The adhered Si atom (and the Si atom denoted by a square in Figure 8a,b) was moved step by step to produce the potential $U^{\prime }$, and the configurational integral was obtained by Equation (21) with $N_{0}$ = 25. As shown in Figure 8c, the FEZ and FEA decreased with the temperature up to 2000 K, while the DFE increased slightly from −0.022 eV up to 0.056 eV. In the temperature range from 200 K to 580 K, the DFE as less than 0.01 eV, and the DFE equaled zero for 400 K. According to the thermal dynamics, the probability for the future deposited atoms adhering to either the zigzag or the armchair edge was equal when the substrate was kept at 400 K, which should be the most optimum temperature for the silicene grain growing to form a continuous graphene-like structure. In previous experiments [3,27], the optimum temperature was 493–580 K, which was about one hundred Kelvin higher than our prediction. The difference may have resulted from the limited computational precision of DFT. For calculating $U_{0}$ and $U^{\prime }\left(0\ldots x_{i}^{\prime }\ldots 0,X^{3M}\right)$ in Equation (13) and (16), it is well known that the calculation results of DFT depend significantly on the specifically employed basis sets and exchange-correlation functionals of the electron density, for which the recent work [45,46] might provide better choices.

## 5. Summary

In summary, DIA was developed to calculate the free energy (or PF) of 2D materials on a substrate, and the high calculation precision was validated by MD simulations. It should be pointed out that such a test is much more stringent than the comparisons between the results derived from the PF and experiments because the same interaction potential is used in both calculations of the PF and the MD simulations, while the potential may not correctly describe the realistic interactions between atoms concerned in the experiment. As for the efficiency, DIA works at least four orders of magnitude faster than the most efficient method, ANS, developed previously, and enables calculations of the free energy based on ab initio calculations to predict the optimal conditions for novel 2D materials’ growth on a substrate, which would greatly shorten the way to experimental realization.
